# Dynamics of laser-induced tunable focusing in silicon

**DOI:** 10.1038/s41598-022-10112-3

**Published:** 2022-04-15

**Authors:** Nadav Shabairou, Maor Tiferet, Zeev Zalevsky, Moshe Sinvani

**Affiliations:** Faculty of Engineering and the Nano-Technology Center, Bar-Ilan University, 52900 Ramat Gan, Israel

**Keywords:** Super-resolution microscopy, Ultrafast photonics

## Abstract

We report here on focusing of a probe IR (λ = 1.55 μm) laser beam in silicon. The focusing is done by a second pump laser beam, at λ = 0.775 μm and 30 ps pulse width, with a donut shape that is launched collinearly and simultaneously (with some delay time) with the IR beam pulse. The pump beam pulse is absorbed in the silicon and creates, temporally, a free charge carriers (FCCs) donut pattern in the silicon. Following the plasma dispersion effect, the donut FCCs shapes a complex index of refraction pattern in the silicon that serves as a sort of dynamic GRIN lens for the probe beam due to the diffusion of the FCCs towards the donut center. This lens can be tuned to its focal point by the pump-probe delay time to reduce the point spread function (PSF) of the IR probe beam. We start seeing the focusing of the probe beam at pump-probe delay time of $\mathrm{\Delta t }\approx 100\mathrm{ ps}$. The best focusing (results in PSF $< 2\mathrm{ \mu m}$) was observed at $\mathrm{\Delta t}\approx 350\mathrm{ ps}$ and it slowly degrades before the FCCs full recombination at $\mathrm{\Delta t }\sim 12\mathrm{ ns}$. We propose this beam shaping method to overcome the diffraction resolution limit in silicon microscopy on and deep under the silicon surface dependent on the pump wavelength and the pulse width. We also proposed this technique for direct measurement of the FCCs dynamics.

## Introduction

The limitation on the spatial resolution in optical microscopy due to the diffraction property of light was discovered by Ernst Abbe in 1873. According to Abbe's law, the best resolution that can be achieved in an optical microscope cannot be better than half of the wavelength of the illuminating light used in the imaging system. Since then, a great effort was invested to break the diffraction limit barrier and many methods were developed for this purpose. In the past two decades, different methods were proposed to overcome the diffraction limit and to reach a super resolution^1–3^ especially in the field of fluorescence microscopy. One of the most famous and breakthrough methods is the stimulated emission depletion (STED) microscopy developed by S. W. Hell^4–6^, with a proven ability for super-resolution of ~ 20 nm in biological samples and ~ 5 nm for color centers in diamond crystals^7,8^, which is more than a factor of 10 below the diffraction limit in optical microscopy. The method is based on exploiting the typical characteristics of the fluorescent molecules used for staining the sample. The molecules is excites by a pulsed laser beam focused on the sample with optimal Gaussian shape. A donut shape second pulsed laser beam, functions by stimulating depleting fluorescence laterally around the center of the excitation beam spot on the sample, while leaving in the center an active focal spot to emit fluorescence. This sharpened the PSF of the excitation beam, while allowing spatial control of the emission from the sample, which results to enhanced resolution.

Some other methods for super-resolution in fluorescence microscopy were introduced as well, such as photoactivated localization microscopy (PALM)^9,10^ and stochastic optical reconstruction microscopy (STORM)^11^.

There are other methods proposed for nanoscopy, based on pump–probe techniques where the pump is in a donut form that shapes the probe beam exploiting different physical mechanisms. However, they all apply on the surface and need either an extra photochromic layer to be added on the sample surface^12^, various types of nanoparticles to be added on the imaged surface^13^, or scanning with 3 collinear laser beams^14^.

In this paper, we introduce tunable focusing of IR laser beam in bulk silicon, where a donut-shaped pump beam focuses the IR probe beam with no need for an extra agent as proposed in our previous papers^15,16^. It may, also, allows imaging on and deep under the silicon surface, depending on the pump wavelength and its pulse duration, and can be especially useful for silicon microscopy^17,18^. The result of our technique is narrowing or sharpening the PSF of the IR probe beam. Therefore, spatial frequencies higher than the ordinary diffraction-limited Gaussian shape PSF are induced in a similar way as it is done in STED microscopy, but of course, by a completely different physical mechanism—the PDE.

### Plasma dispersion effect (PDE)

The physical mechanism we use for the modification of the silicon refractive index is the PDE, in which a change in the concentration of the FCC in the silicon modifies its complex refractive index. The complex refractive index can be expressed as $\mathrm{n}={\mathrm{n}}_{\mathrm{r}}+\mathrm{ ik}$ where the real part is the conventional index of refraction ${\mathrm{n}}_{\mathrm{r}}$. The imaginary part $\mathrm{k}$ is the optical extinction coefficient and it is related to the absorption coefficient $\mathrm{\alpha }$, by the equation $\mathrm{k}=\mathrm{\alpha \lambda }/4\uppi$. The PDE change of the real refractive index $\mathrm{\Delta n}$, and the absorption coefficient, Δα can be predicted by the free-carrier theory (Drude model)^19^:

1 $$\mathrm{\Delta n}=\left(\frac{{-\mathrm{e}}^{2}{\uplambda }_{0}^{2}}{8{\uppi }^{2}{\mathrm{c}}^{2}{\upvarepsilon }_{0}\mathrm{n}}\right)\cdot \left(\frac{\Delta {\mathrm{N}}_{\mathrm{e}}}{{\mathrm{m}}_{\mathrm{ce}}^{*}}+\frac{\Delta {\mathrm{N}}_{\mathrm{h}}}{{\mathrm{m}}_{\mathrm{ch}}^{*}}\right)$$

2 $$\mathrm{\Delta \alpha }=\left(\frac{{\mathrm{e}}^{3}{\uplambda }_{0}^{2}}{4{\uppi }^{2}{\mathrm{c}}^{3}{\upvarepsilon }_{0}\mathrm{n}}\right)\cdot \left(\frac{{\Delta \mathrm{N}}_{\mathrm{e}}}{{\upmu }_{\mathrm{e}}{{(\mathrm{m}}_{\mathrm{ce}}^{*})}^{2}}+\frac{\Delta {\mathrm{N}}_{\mathrm{h}}}{{\upmu }_{\mathrm{h}}{\left({\mathrm{m}}_{\mathrm{ch}}^{*}\right)}^{2}}\right)$$

where $\mathrm{e}$ is the electron charge, $\mathrm{c}$ is the velocity of light in vacuum, ${\upmu }_{\mathrm{e}}, {\upmu }_{\mathrm{h}}$ are the electron and hole mobility respectively, ${\mathrm{m}}_{\mathrm{ce}}^{*}, {\mathrm{m}}_{\mathrm{ch}}^{*}$ are their effective masses, ${\mathrm{\Delta N}}_{\mathrm{e}} ,\Delta {\mathrm{N}}_{\mathrm{h}}$ are their concentrations, ${\upvarepsilon }_{0}$ is the free space permittivity and ${\uplambda }_{0}$ is the free-space wavelength.

For c-Si and probe beam at wavelength, $\uplambda =1550\mathrm{ nm},$ Soref and Bennet^19,20^ extracted empirical formulas for $\mathrm{\Delta n}$ and $\mathrm{\Delta \alpha }$ as a function of the electron and holes concentration ${\mathrm{\Delta N}}_{\mathrm{e}}\mathrm{ and \Delta }{\mathrm{N}}_{\mathrm{h}}$ respectively.

3 $$\mathrm{\Delta n}=-8.8\bullet {10}^{-22}\bullet {\mathrm{\Delta N}}_{\mathrm{e}}-8.5\bullet {10}^{-18}\bullet {\mathrm{\Delta N}}_{\mathrm{h}}^{0.8}$$

4 $$\mathrm{\Delta \alpha }=8.5\bullet {10}^{-18}\bullet\Delta {\mathrm{N}}_{\mathrm{e}}+6.0\bullet {10}^{-18}\bullet\Delta {\mathrm{N}}_{\mathrm{h}}$$

In our experiment, the pump laser pulses temporally generate the electron and holes concentration where each photon excites an electron–hole pair via lattice phonon assistance. This inter-band photo-excited electron–hole pair behaves as a quasi-particle that is bounded by Coulomb attraction and is free to move in the semiconductor. Since this pair is electrically neutral it does not drift under an electric field. The pair can move only due to diffusion from high-density to low-density regions in the semiconductor^21,22^. This ambipolar diffusion of electron–hole pairs is slower than that of separate electrons or holes. According to Eqs. (1) and (2), the increase in the FCCs concentration causes a decrease in the real refractive index $\mathrm{\Delta n},$ and an increase in the absorption $\mathrm{\Delta \alpha }$ that attenuates the IR beam in the silicon.

Silicon microscopy, which is based on the FCCs absorption in silicon, plays an important role in imaging and probing techniques for inspection and metrology in the engineering process of silicon integrated circuits (ICs). The main tool in this technology is an IR laser beam acting as a contactless probe, applied on the IC from the backside while the IC under operation, is allowing a direct access to the active areas buried inside the silicon chip^18,23^ for electrical measurement and imaging. This technology can be used, also, for optical reading of nonvolatile memories on a chip^24^ and contactless probing of secret (cryptography) data on field programmable gate arrays^25^. Today, where the ICs production technology is around 10 nm in size, there is a great need for super-resolution in silicon microscopy. Our method for super-resolution that is described in this paper, may play an important role in sharpening this probe well below the diffraction limit. It may advance silicon microscopy in general and failure analysis in particular, towards super-resolution as the STED technique did for fluorescence microscopy.

Another aspect that can be studied by our technique is the dynamics of FCCs excited by picosecond laser pulses. The diffusion and lifetime can be studied by direct measurement of the IR probe laser transmission in the silicon. In this work, we start seeing the focusing of the probe beam at pump–probe delay time of $\mathrm{\Delta t }\approx 100\mathrm{ ps}$. The best focusing is observed at pump–probe delay time of $\mathrm{\Delta t }\approx 350\mathrm{ ps}$ and it degrades before the FCCs full recombination at $\mathrm{\Delta t }\sim 12\mathrm{ ns}$. The use of shorter pulse width for the pump laser with a lateral donut shape allows us to reduce the probe beam PSF’s full width at half maximum (FWHM), PSF < 2 μm. We show here, to our best knowledge, for the first time the tunable focusing of an IR laser beam inside bulk silicon and direct measurement of the dynamics of picosecond laser induced FCCs in silicon.

## Results

### The concept

**Figure 1:**
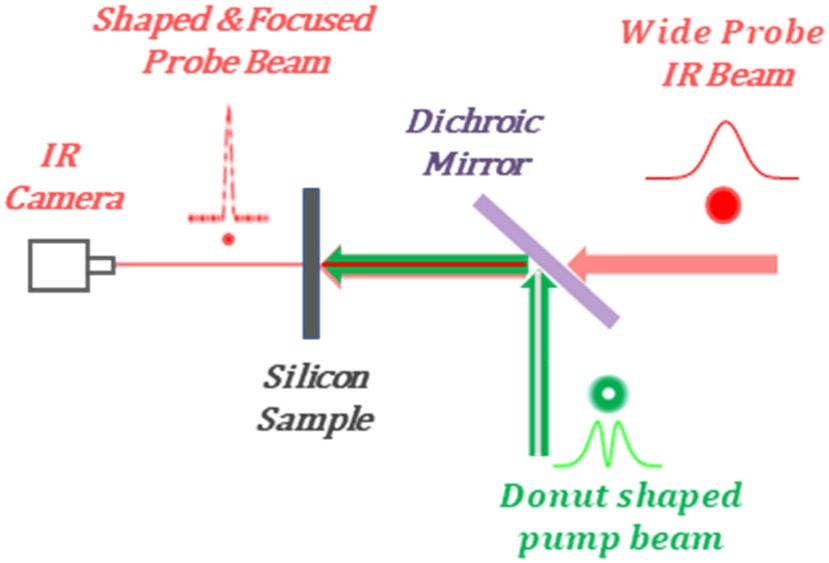
The concept of IR beam focusing inside the silicon. A donut shaped pump laser pulse is absorbed in the silicon and induces, temporally, a donut shape FCCs area with the same size, in the silicon. A second IR probe laser pulse that is lunched right after, is being blocked by the FCCs donut area via FCCs absorption. The center of the IR beam is transmitted and focused to a narrow beam via FCCs dispersion.

**Figure 2:**
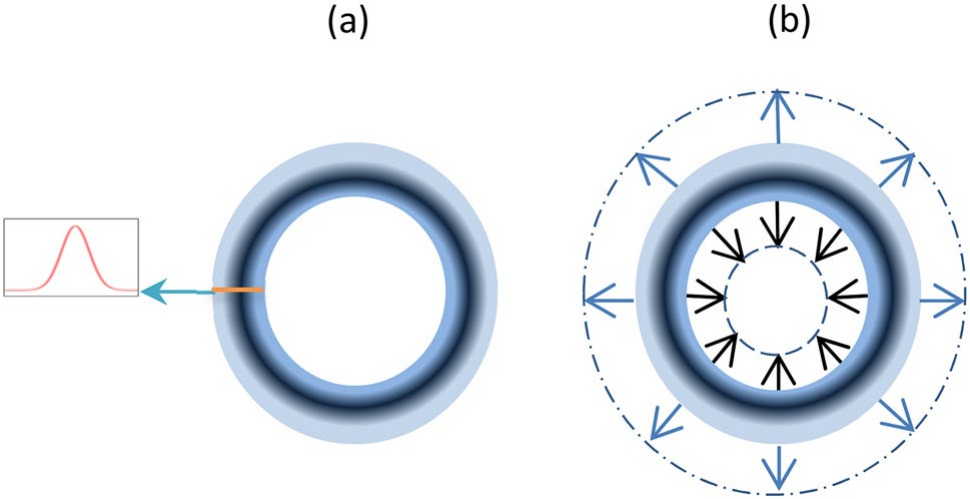
The FCCs pattern induced by the pump beam and its dynamics. Illustration of the donut pattern of the FCCs that is excited by the donut shaped pump beam on the silicon is shown in (**a**), in the insert we show the Gaussian distribution of FCCs density across the donut. In (**b**) we show the diffusion directions of the FCCs; out of the donut (blue arrows), the part of the probe beam that is outside the donut will defocus while toward the donut’s center (black arrows) a focusing will occur for the part of the probe beam enclosed in the inner part of the donut. The optical axis is perpendicular to the page.

A schematic description of our concept is shown in Fig. 1. An IR laser probe pulse in silicon can be shaped by changing the silicon complex refractive index spatially and temporally via a second laser pump pulse, with a donut shape, that absorbed in the silicon. According to the PDE, this change is induced by the change in the FCCs density in the silicon due to the absorbed pump pulse, where each absorbed photon excites one electron–hole pair with lattice phonon assistance. The idea for the IR beam shaping, which we first proposed in our previous work^15^ and fully demonstrated firstly in this paper, is based on the use of a donut shape for the pump beam as illustrated in Fig. 1. The IR probe laser pulse hits the silicon, collinearly and simultaneously (with some delay time that will be discussed below) with the donut-shaped pump laser pulse. The pump pulse creates, temporally, a donut pattern of FCCs in its spot size in the silicon, as illustrated in Fig. 2a.

**Figure 3:**
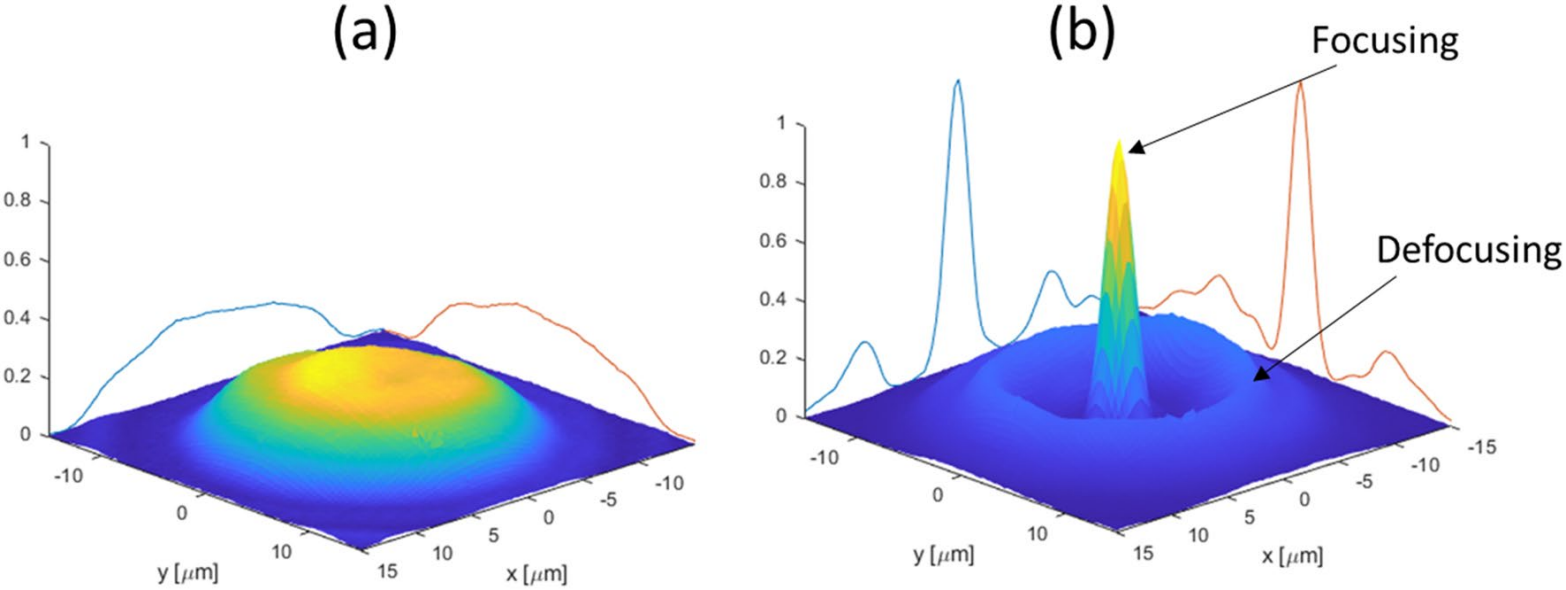
Focusing demonstration. Images of the probe beam’s PSF in the silicon are shown, without the pump (**a**), and with the pump beam (**b**). The trace of the donut ring of the pump laser spot in the silicon is clearly seen in (**b**) as a groove of $\sim$ 5 µm radius that blocks the probe IR beam completely via the FCCs absorption. Inside the donut, one may see the focused part, and outside of it is the defocused part of the IR probe beam. The practical result, in this case, is a narrower PSF probe’s spot which is narrowed by a factor of $\sim$ 7 and became higher by a factor of more than 2. The wavelength of the pump beam is 0.775 µm and the IR probe beam is 1.55 µm. The pulse repetition rate is 100 kHz.

The probe beam hits the donut’s distributed FCCs pattern in the silicon and is shaped by it as follows: Part of the probe beam’s PSF is blocked, via absorption, by the higher FCCs density area on the donut ring. The rest of the probe beam disperse radially in two directions: (1) inside the donut—focused towards its center, and (2) outside the donut—defocused outside of it. The diffusion dynamics of the FCCs donut pattern towards its surroundings in the silicon are illustrated in Fig. 2b. To control the dynamics of the FCCs in a high-resolution time scale, we use in this work picosecond lasers with a pulse width of 30 ps for the pump beam and 50 ps for the probe beam. This is compared to the 17 ns pulse width pump laser in our previous work^15^, which masks the focusing due to the FCCs diffusion during the wide pump pulse. According to PDE, the index of refraction has the same dynamics as the FCCs. Therefore, the hole in the FCCs donut’s pattern acts as a sort of a dynamic GRIN lens, that its index of refraction profile may be governed by two parameters. First, the diffusion dynamics of the FCCs pattern towards the donut center can be tuned by the delay time of the probe beam pulse after the pump beam pulse. Second, the diameter of the donut pump beam in the sample can be manipulated by the change of the vortex plate topological order. An experimental demonstration of the focusing and defocusing process is shown in Fig. 3. Also, the focal length depends on the FCCs concentration, it become shorter as the FCCs concentration increases by the increase of the pump pulse intensity.

### The effect of the donut diameter on the focusing

**Figure 4:**
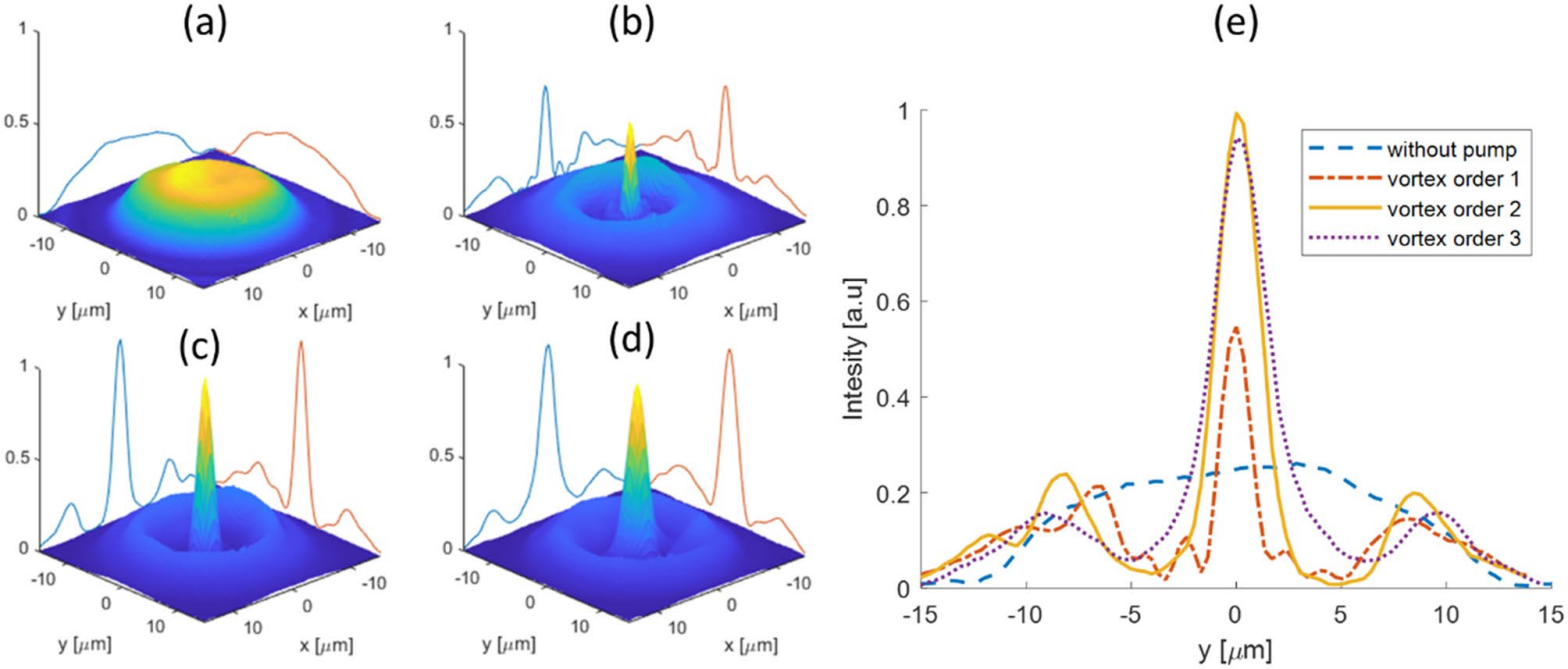
The effect of donut pump diameter on the IR probe beam focusing. Images of the probe beam, without pump beam (**a**), and with donut pump with vortex orders 1, 2, and 3 applied on the pump beam are shown in (**b**)–(**d**), respectively. In (**e**) are shown the profiles of the probe beam under the different vortex orders for the donut pump beam. The best focusing is in vortex order 1. For higher vortex orders, the probe PSF is wider moderately but stronger. The blue curve is the probe PSF without the pump beam, and one can see the dramatic improvement in the PSF of the probe beam with the application of the donut-shaped pump beam.

**Figure 5:**
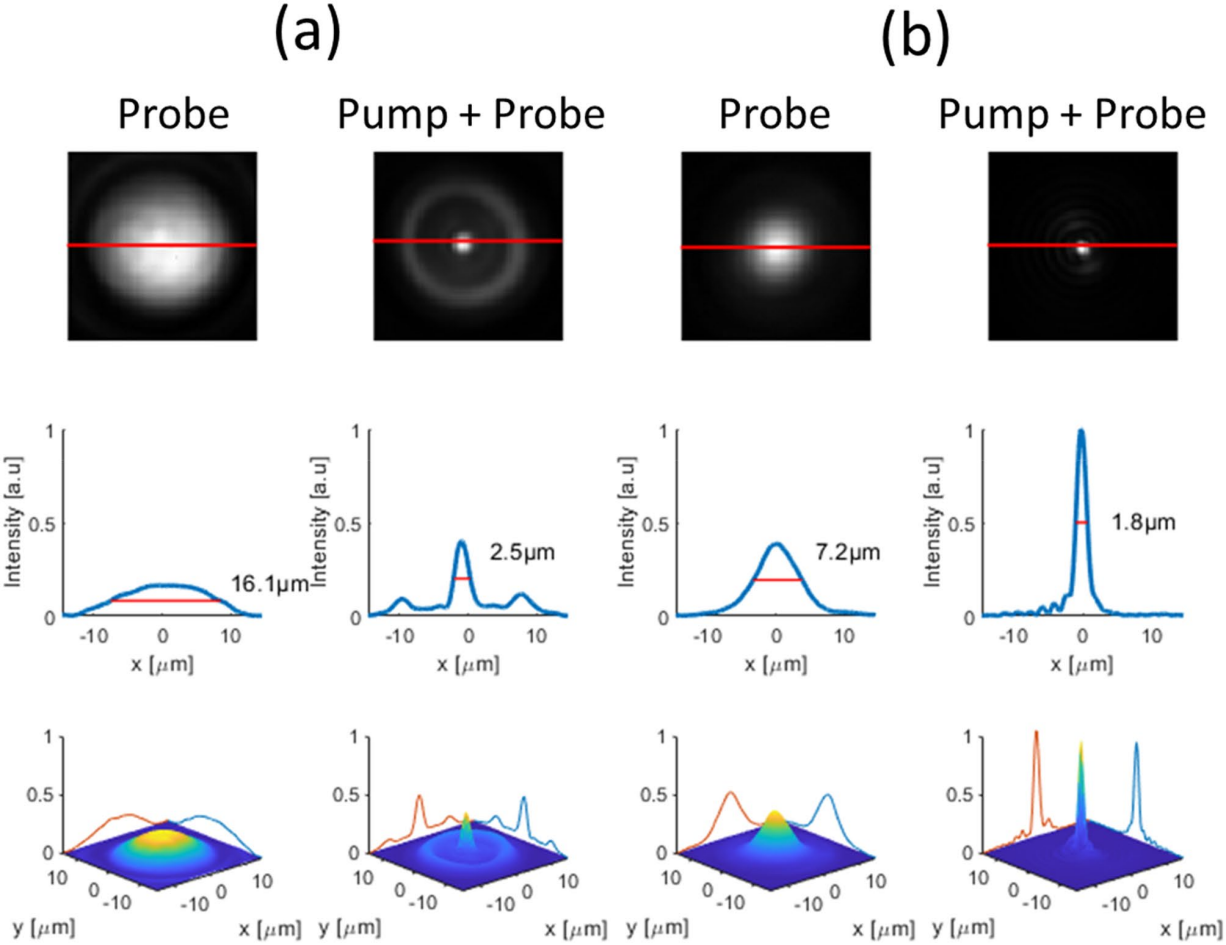
The effect of the relative diameter of pump-probe laser spots on the focusing. In the first row, we present the probe images generated in the sample with and without the effect of the pump, in two cases: (**a**) where the spot diameter of the probe is bigger than the pump’s spot diameter and (**b**) where the pump spot diameter is smaller than the probe beam’s diameter. On the second and third rows, we present the profile and the 3-D images, for each image, respectively.

In Fig. 4. we demonstrate the focusing of the probe beam under the pump beam with three different diameters of the donut beam. To convert the lateral shape of the pump beam, from Gaussian to donut shape we use a diffractive optical vortex element^26,27^. The donut shape of pump diameter depends on the topological order of the vortex plate, where the diameter increases for higher orders. We use the three first topological orders of the vortex plate to change the pump donut’s diameter (see Supplementary 1 on Vortex phase plate principle of operation). We can estimate the donut pump beam radius from Fig. 4e to be 3.7, 5, and 6 μm for vortex orders 1, 2, 3 respectively. The effect of the relative size of the pump–probe laser's spots on the focusing is demonstrated in Fig. 5.

As mentioned above, the focusing phenomenon we observe is due to the GRIN-like lens, generated by the spatial distribution of the FCCs and consequently the distribution of the refractive index inside the donut area induced in the silicon by the pump beam. However, the spatial distribution of the refraction index is dynamic in time due to the ambipolar diffusion of the FCCs.

### Time evolution of the IR probe focusing due to FCCs diffusion

**Figure 6:**
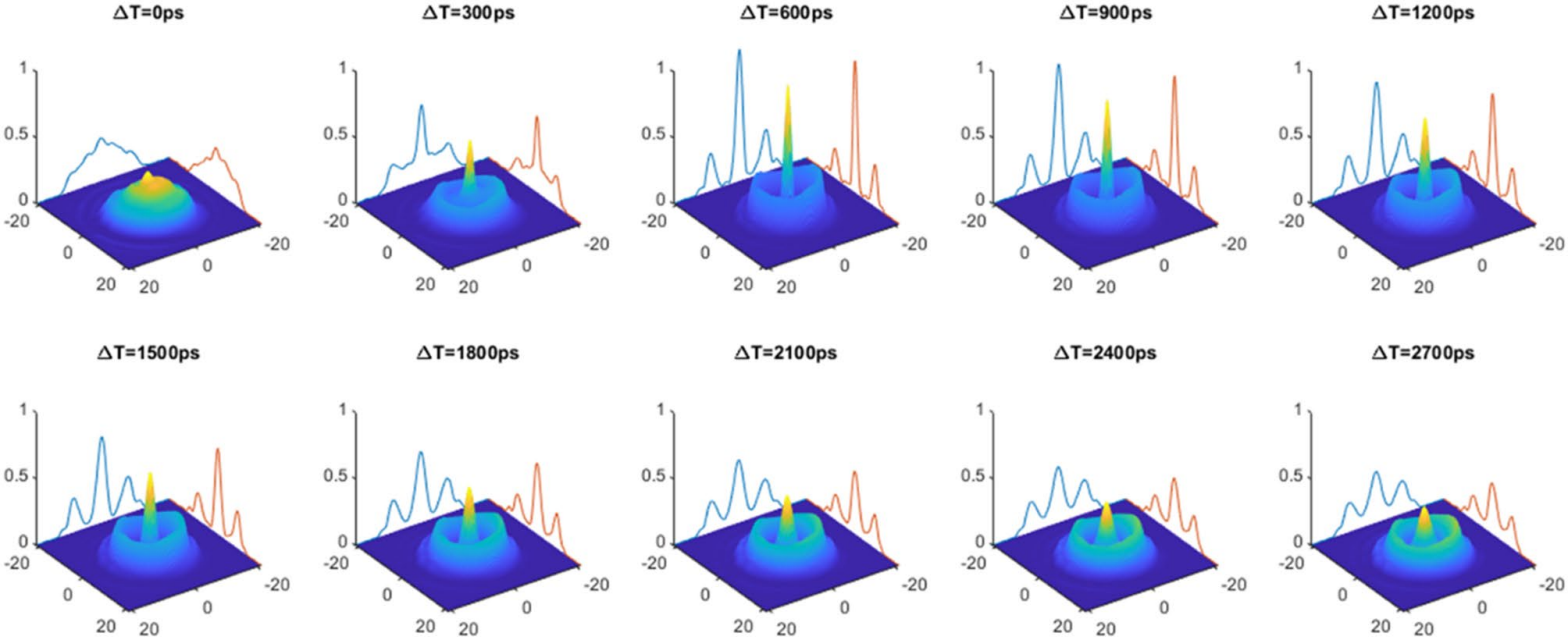
The focusing dynamics. Here is a sequence of 3-D images of the probe IR beam inside the silicon sample as taken with the InGaAs camera vs the pump–probe delay time. The best focusing we get in this experiment appears at pump–probe delay time of $\sim$ 600 ps, and the absorption depth ring (the trench around the focusing peak) gets its maximum on the same delay time. The X, Y axis are in μm, and the pump donut beam is shaped by vortex order 2. The laser pulse repetition rate is 40 kHz.The optical axis is in the Z direction.

A sequence of images for the shaped probe beam in the silicon sample for various pump–probe delay times, Δt, are shown in Fig. 6. One can see that the focusing peak increases rapidly with time, where its sharpest value is at Δt = 600 ps and then it decreases slowly due to the FCC diffusion and it is completed with the FCCs recombination process at Δt $\sim$ 12 ns (see Supplementary Movie 1 that shows the dynamics of the focusing for long pump–probe delay times).

**Figure 7:**
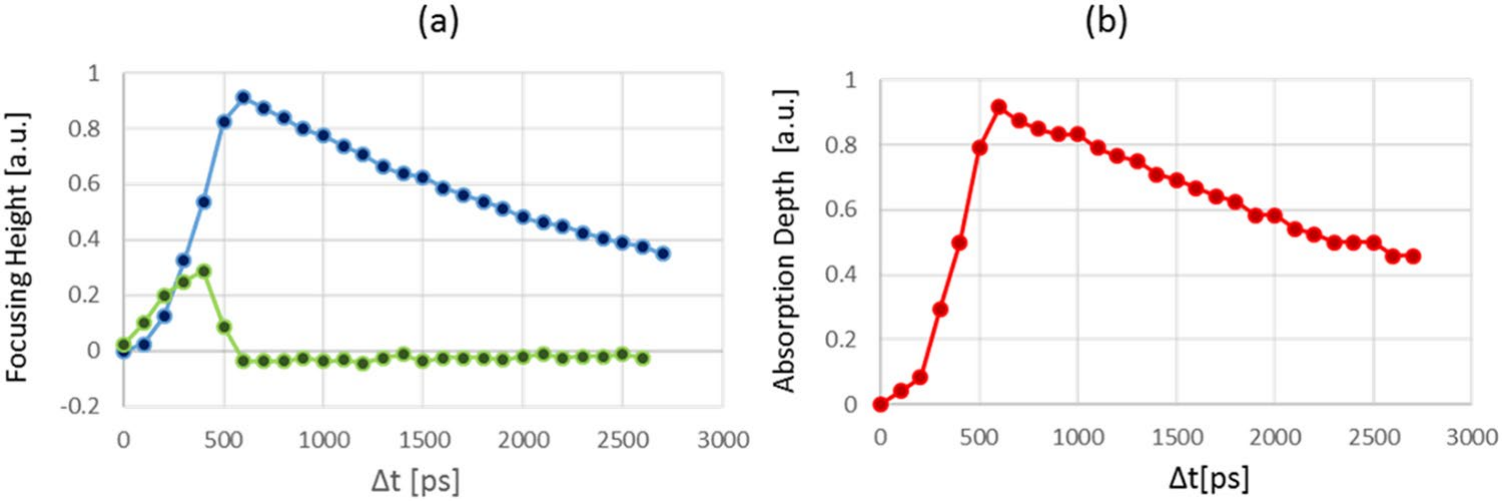
Time evolution analysis of the FCC focusing (**a**) and absorption (**b**). It was taken from the same data as in Fig. 6. In (**a**) are shown the focusing peak’s height (blue) and its derivative (green) versus the pump–probe delay time. In (**b**) is given the donut ring absorption depth seen in Fig. 6 that represents the absorption of the probe beam by the FCCs donut shape that is induced by the pump beam.

Also, concomitantly, the FCCs absorption that appears as a donut groove around the focusing peak (as explained above) is rapidly increasing with the delay time to its maximum value at $\mathrm{\Delta t}=600\mathrm{ ps}$, and then decreases slowly due to FCCs diffusion and recombination. Analysis of the images for the whole sequence where Δt is taken in increments of 100 ps (see Supplementary Movie 2 that shows the dynamics of the focusing) are given in Fig. 7. The values for the focusing peak and the absorption depth were taken after subtraction of every image in the sequence from the initial image of the probe beam. Where the initial image was taken 200 ps befor the pump beam was applied at Δt = 0. We see in Fig. 7 that the focusing height and the absorption depth have similar dynamics with maximum value at $\mathrm{\Delta t}=600\mathrm{ ps}$ and they represent the change in the refractive index Δn and the change in the absorption Δα, respectively, according to the PDE theory. We also see two distinct different behaviors for the two Δt regions below $\mathrm{\Delta t }= 600\mathrm{ ps}$ and above it. In the first region it is clearly seen in Fig. 7b that the FCCs absorption depth builds up with time and gets its maximum (full absorption of the probe) at 600 ps to quasi-equilibrium state. The FCCs concentration gets its maximum value on the donut ring pattern and then the slow diffusion of the FCCs appears, both are clearly seen on the derivative of the focusing peak plotted by the green line in Fig. 7a. It can be seen, also, at the images in Fig. 6 as a donut shape blocked area build up around the focusing peak. We know from the literature that hot carriers induced by very fast laser pulses equilibrate and thermalize with the crystal lattice phonons within few pico-seconds^28–30^. Therefore, we attribute the increase of the focusing peak on the first 500 ps to the FCCs build up in the silicon. This long FCCs build up time is due to the duration of the laser pulses for both the pump and the probe beams, the individual jitter of each laser and the jitter of the time delay generator we use to synchronize in between the lasers. This is approved by the calculation of the convolution product of these parameters. In the second region, we suppose that the dominant process is FCCs ambipolar diffusion^21,22,31,32^. We try to extract the diffusivity from time evolution of the width (FWHM) of the focusing peak, assuming a Gaussian shape^21,22^, and the value we get is D_a_ ≃ 3 cm^2^/s which is similar to the value of D_a_ = 2.6 cm^2^/s that measured by Mouskeftaras et al^32^. It has to be emphasized here that the focusing width has a low sensitivity for the pump–probe delay time. However, it can be seen from Fig. 7a that the height of the focusing peak is clearly more sensitive to the pump–probe delay time expressed via smooth monotonic decrease. Therefore, we suggest using the height of the focusing peak as a better parameter to measure the dynamics of the FCCs. This needs more investigation and it will be discussed in our future research.

### Jitter free experiment

**Figure 8:**
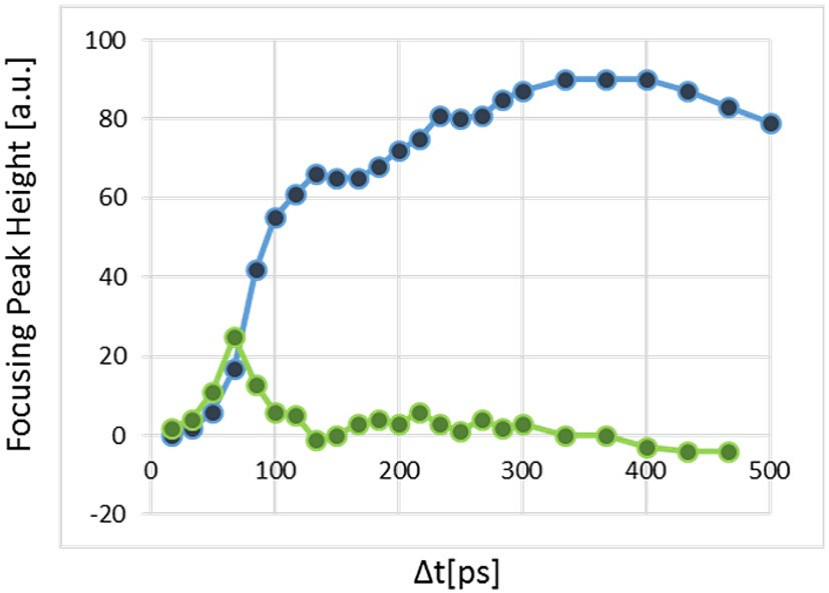
Jitter free experiment. The time evolution of the focusing peak height (blue curve) and its derivative (green curve) on time-region below 500 ps (the first region) are shown. The 30 ps pump pulse applied at Δt = 0. The sharp rise at the first 100 ps is due to FCCs build-up induced by the pump pulse. Above 100 ps the increase in the focusing peak height is due to the slow ambipolar diffusion of the FCCs towards the donut center where the best focusing appears at Δt ≈ 350 ps.

To reduce the problem of the laser’s pulse width and the jitter, to allow monitoring the FCCs dynamics below 600 ps, we performed the experiment with one laser using its two harmonics. The first harmonic at λ = 1.55 μm with pulse duration of 30 ± 10 ps as a probe beam, and its second harmonic at λ = 0.775 μm as a pump beam. In this case, the pump–probe delay time setting was done by an optical delay line. This solves the pump-probe jitter problem. The results of this experiment are given in Fig. 8 (see Supplementary Movie 3 that shows the dynamics of the focusing in this case). The sharp rise of the focusing peak that represents the FCCs build-up, was reduced to ~ 100 ps. This result agrees with the convolution calculation between the pump and the probe beams’ pulses. After 100 ps the focusing peak increases slowly, due to the FCCs ambipolar diffusion, to its highest value at Δt ≈ 350 ps where in this pump–probe delay time the best focusing is achieved, see Fig. 8. The FCCs distribution and therefore the index of refraction distribution inside the donut area at this time creates a sort of a GRIN lens, at its focal point, as explained above. For Δt values higher than ≈ 400 ps the focusing peak’s height decreases slowly, almost at the same pace as shown on the second region in Fig. 7a.

### Numerical simulation and focusing inside the silicon

We developed a numerical simulation to support our model for analysis of the focusing parameters. Our simulation is based on three main steps. The first step was to calculate the spatial pattern of the complex index of refraction that is generated in silicon by the pump beam. The pump beam’s intensity distribution was modeled by using the analytic equation of the Laguerre–Gaussian beam (vortex equation)^26^. The distribution of the pattern of the pump photons, which are absorbed inside the silicon, was calculated according to intensity loss along the light propagation axis in the silicon (Bear–Lambert law). We assumed a quantum efficiency of 1, where each pump photon generates 1 electron–hole pair that is free to move in the silicon as an exciton by an ambipolar diffusion. According to PDE the FCCs distribution pattern, which is created by the pump photons, is translated to a change in the refractive index Δn, and the absorption coefficient Δα, according to Eqs. (3) and (4) respectively.

**Figure 9:**
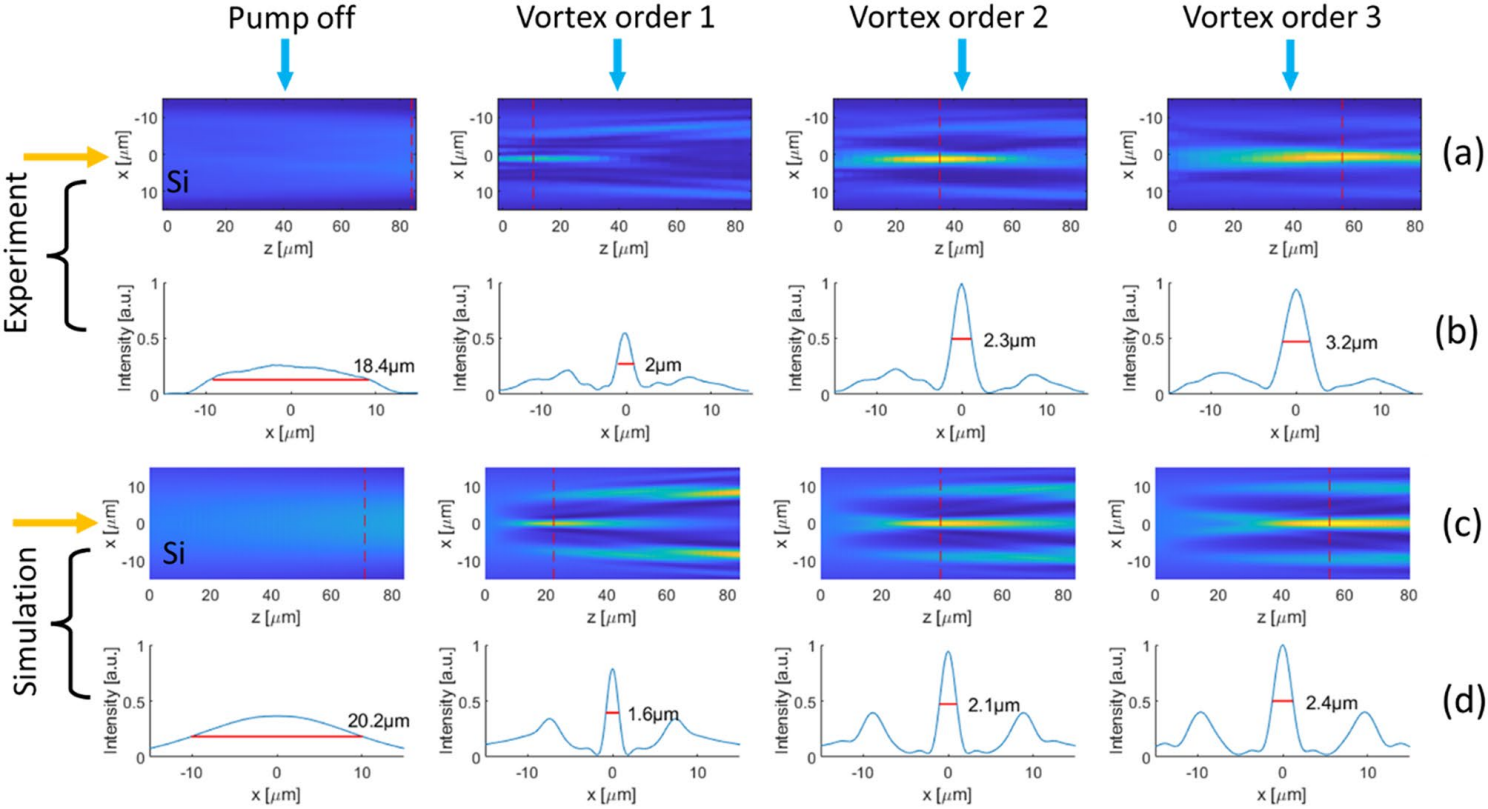
Experimental and simulation results for probe’s focusing inside the silicon. The experimental results are compared to the simulation results and are shown for the shaped probe beam. The lasers illuminate the silicon sample from left to right as indicated by the yellow arrows. In rows (**a**) and (**c**) we show Images of the trajectories of the probe beam inside the silicon in 4 cases. In the first case without the pump beam (the left column) and in the other three cases the pump beam applied in three different vortex’s topological orders 1, 2 and 3. In rows (**b**) and (**d**) we show the profile of the probe beam at the focal point (indicated on each image by a dashed vertical red line) in the four cases. The simulation results are in good correlation with the experimental results.

The second step is the calculation of the diffusion of the FCCs that are generated in the silicon, for a pump-probe delay time of Δt = 350 ps, where we get the best focusing in the experiment as seen in Fig. 8. The diffusion was simulated according to the diffusion equation:

5 $$\frac{\partial \mathrm{p}\left(\mathrm{r},\mathrm{t}\right)}{\partial \mathrm{t}}=\mathrm{D}\bullet {\nabla }^{2}\mathrm{p}\left(\mathrm{r},\mathrm{t}\right)-\frac{\mathrm{p}\left(\mathrm{r},\mathrm{t}\right)}{\uptau }$$

where $\mathrm{p}(\mathrm{r},\mathrm{t})$ is the FCCs concentration, D is the diffusion coefficient and τ is the effective recombination time. We neglected the heating effect occurring in the sample due to the pump pulse and its impact either on the diffusion coefficient or the index of refraction (we set the diffusion coefficient to be constant at room temperature). By simple thermodynamic calculation, we see that the pump pulse increases the induced FCCs pattern by only several Celsius degrees. The simulation was done on a 2-dimensional slice in the XZ plane along the center of the beam (r,z), assuming radial symmetry in the XY plane. At the third and last step, we simulated the propagation of the probe beam according to the temporal distribution of the FCCs in the silicon. The simulation was performed by using the Finite-Difference Frequency-Domain (FDFD) method^33^. In Fig. 9 we demonstrate the simulation results comper to the experiment and we see good correlation.

## Discussion

As mentioned above, the FCCs induced by the pump pulse modify the complex index of refraction in both of its parameters Δα and Δn. Note that similar pump-probe experiments in semiconductors^21,22,31,32^ are related to the FCCs absorption Δα, only, when investigating the photo exited FCCs dynamics in silicon. In our experiment, we see also, the effect of FCCs dispersion that allows us to demonstrate defocusing of IR probe beam when applying Gaussian shape pump beam^15^, and to demonstrate focusing of probe beam when a donut shape pump beam is applied. To get the focusing we have to use a very short laser pulse to govern the diffusion of the FCCs induced by the donut pattern, which is occurring towards the donut’s center, and to get the “GRIN lens” like, distribution of the refractive index inside the FCCs donut pattern.

In this work, we use a pump laser with pulse width of τ = 30 ps and λ = 0.775 μm while in our previous publication we used pump laser with τ = 17 ns, and λ = 0.532 μm^15^. The shorter pulse width for the pump beam, shorten the FCCs diffusion length and allowed the focusing to sharpen the PSF of the IR probe beam. The penetration depth of the pump beam in silicon increases for longer wavelengths and it allows focusing of the probe beam inside the silicon. The use of wavelength of λ = 0.775 μm, that has a penetration depth of 10 μm (compared to 1 μm at λ = 0.532 μm), for the pump beam allows the shaping of the probe beam deeper in the silicon.

The focusing we achieved in this paper yielded PSF’s FWHM of $\sim$ 1 μm. We cannot get better focusing with the present experimental setup because of dispersion of the single objective lens that we used for both the pump and the probe beams. We believe we can reduce the PSF considerably, with better objective lenses existing in silicon industry today. To get better super-resolution in silicon we have to have the ability to separately focus each beam, the pump and the probe to its optimum. It is important to emphasize here, that the focusing effect we report in this research does not depend on the doping level of the silicon. In preliminary experiments, we get the focusing phenomenon on heavily doped silicon (3 × 10^18^ cm^−3^), and in 20 μm thinned silicon sample also. This information is very important for the usage of our sharpen probe for super-resolution in silicon microscopy for ICs industry.

Our technique can also be used for focusing high-density micro-plasma for writing waveguides and nano-electronics devices inside the silicon which is necessary in the evolving field of integrated opto-electronics^34^. The dynamics of the focusing as demonstrated in Figs. 7 and 8, shows slow ambipolar diffusion (${\mathrm{D}}_{\mathrm{a}}\approx 3\mathrm{ c}{\mathrm{m}}^{2}/\mathrm{s}$) of the FCCs starting from 100 ps and on, till to full recombination occurring at Δt $\sim$ 12 ns. Similarly, Mouskeftaras et al^31,32^ reported on slow ambipolar diffusivity (2.5 cm^2^/s) started from 0.5 ps till 2.5 ns (they used femtosecond laser) in intrinsic silicon bulk sample resembling our samples, and with similar photo-excited FCCs concentrations of $\sim$ 5 × 10^19^ cm^−3^. However, in both experiments (Mouskeftaras^32^ and us) the FCCs were observed directly via PDE and show slow diffusion. Also, in both works the increase in the lattice temperature due to pump pulse is small and do not affect the ambipolar diffusivity. We, also, see slow diffusion in the heavily doped silicon sample and in the 20 μm thick sample we measured. As said above, the dynamics of FCCs will be discussed in our future publication.

## Summery

In this paper, we demonstrated for the first time a new pump–probe technique for tunable focusing of IR probe laser beam in silicon. We showed sharpening of the PSF of the probe beam by a factor of 10 to < 2 μm FWHM, inside the silicon when applying the pump beam. The major parameter for the tuned of the focusing is the pump–probe delay time that monitors the dynamics of the FCCs diffusion. We introduced a physical model to explain the focusing phenomenon with numerical simulation and showed an agreement with the conducted experiments. The IR probe’s focusing technique we develop can be used in the microelectronics industry for failure analysis and tests in ICs. We also show that our technique provides a strong tool for dynamics measurements of band-to-band photo-excited excitons in semiconductors.

## Methods

### Experimental setup

Scheme of the experimental setup is presented in Supplementary 2 Fig. 1. The pump (NKT Onefive KATANA 775 nm, 30 ps pulse width) and the probe [Alphalas PLDD-20M 1550 nm, 50 ps pulse width] lasers synchronized in between them and with the InGaAs camera [FLIR SC2500-NIR] by the delay generator [SRS DG535 Digital Delay Generator]. The Gaussian pump beam was converted to a donut shape by a vortex plate [RPC PHOTONICS VPP-m780] with 8 separate area segments with different topological orders from 1 to 8. The Gaussian probe beam combined with the donut pump beam by the dichroic mirror [Thorlabs DMLP900R] and collinearly directed to the × 20 objective [Mitutoyo M Plan Apo NIR 20x] and focused inside the silicon sample. The InGaAs camera that synchronized with the laser’s pulses, image the probe beam inside the silicon through the × 100 objective [Mitutoyo M Plan Apo NIR 100x]. The diffraction limit resolution of the camera's optics is $\sim$ 2 μm. The maximum pump pulse energy is > 20 nJ. The lasers are linearly polarized diffraction limit beams.

The sample we use is an intrinsic Fz, < 100> c-Si slab with resistivity > 1000 Ω cm, 470 μm thick, and an area of 20 × 20 mm^2^. It was cut from a 4″ silicon wafer optically polished on both sides in parallel.

## Supplementary Information

Supplementary Information 1.

Supplementary Information 2.

Supplementary Video 1.

Supplementary Video 2.

Supplementary Video 3.
