# Supplementary material for: Dynamics of laser-induced tunable focusing in silicon
Source: Sci Rep. 2022 Apr 15;12:6342. doi: 10.1038/s41598-022-10112-3 (PMC9012861; doi:10.1038/s41598-022-10112-3)
Supplement: Supplementary file 1 — Supplementary Information 1. [file 41598_2022_10112_MOESM1_ESM.docx]

Supplementary 1:

Dynamics of Laser-Induced Tunable Focusing

in Silicon

Nadav Shabairou, Maor Tiferet, Zeev Zalevsky and Moshe Sinvani^*^

Faculty of Engineering and the Nano-Technology Center, Bar-Ilan University, Ramat Gan, Israel 52900.

*Corresponding author: [sinvanm@gmail.com](mailto:sinvanm@gmail.com)

Vortex phase plate principle of operation and its topological orders

The optical Vortex phase plate is a unique optic, whose structure is composed entirely of helical or spiral phase steps, whose purpose is to control the phase of the transmitted beam.


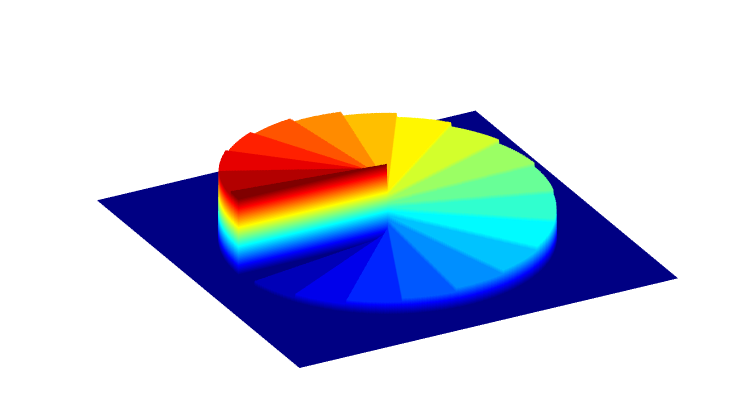


**Figure 1**: Winding “Staircase” surface profile of diffractive vortex phase plate with topological order m=1.

The total etching depth from the top to bottom of “staircase” is a function of the design wavelength and the substrate’s optical index. Generally, this depth is of the same order of magnitude as the design wavelength. Therefore, each optical Vortex phase plate is wavelength specific.

The topological order, denoted in the literature as m, refers to the number of 2π cycles (i.e. “staircases”) that are etched around 360 degree turn of diffractive surface. In Fig.1 above, one “staircase” cycle covers entire 360° turn of surface, so m=1 for that vortex phase plate. In Fig.2 below, the surface profiles are illustrated for higher topological orders Vortex phase plate with m=2, m=3 and m=4.
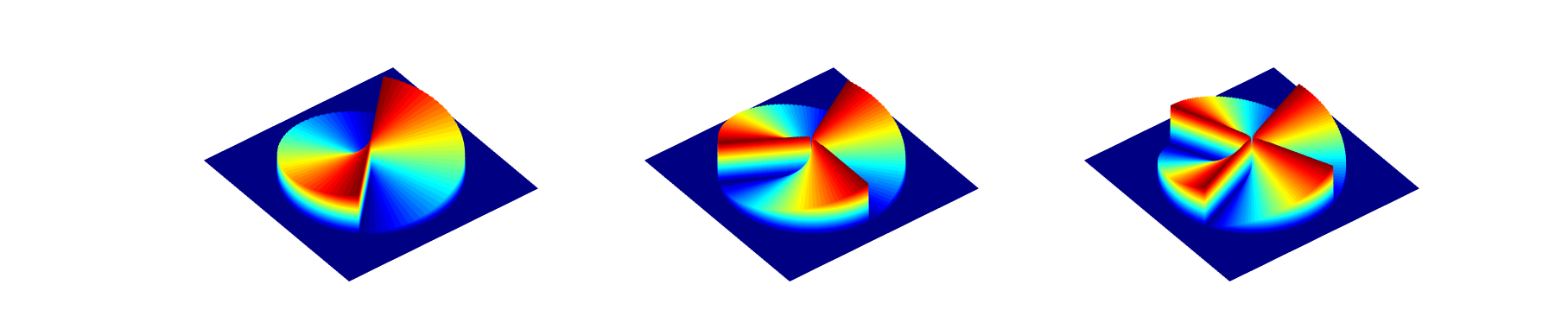


**Figure 2**: Surface profiles for vortex plates with topological orders m=2, m=3 and m=4.

One main effect of a higher topological orders is an increase in the angular moment of the vortex beam by a factor of m. Another effect is to magnify the donut intensity pattern dimensions by a factor of m, as illustrated in the below simulation.

Figure 3**:** Simulated “far-field” intensity images of Vortex beams with variable *m* value**.**
