# Supplementary material for: Dynamics of laser-induced tunable focusing in silicon
Source: Sci Rep. 2022 Apr 15;12:6342. doi: 10.1038/s41598-022-10112-3 (PMC9012861; doi:10.1038/s41598-022-10112-3)
Supplement: Supplementary file 2 — Supplementary Information 2. [file 41598_2022_10112_MOESM2_ESM.docx]

Supplementary 2:

Dynamics of Laser-Induced Tunable Focusing

in Silicon

Nadav Shabairou, Maor Tiferet, Zeev Zalevsky and Moshe Sinvani^*^

Faculty of Engineering and the Nano-Technology Center, Bar-Ilan University, Ramat Gan, Israel 52900.

*Corresponding author: [sinvanm@gmail.com](mailto:sinvanm@gmail.com)


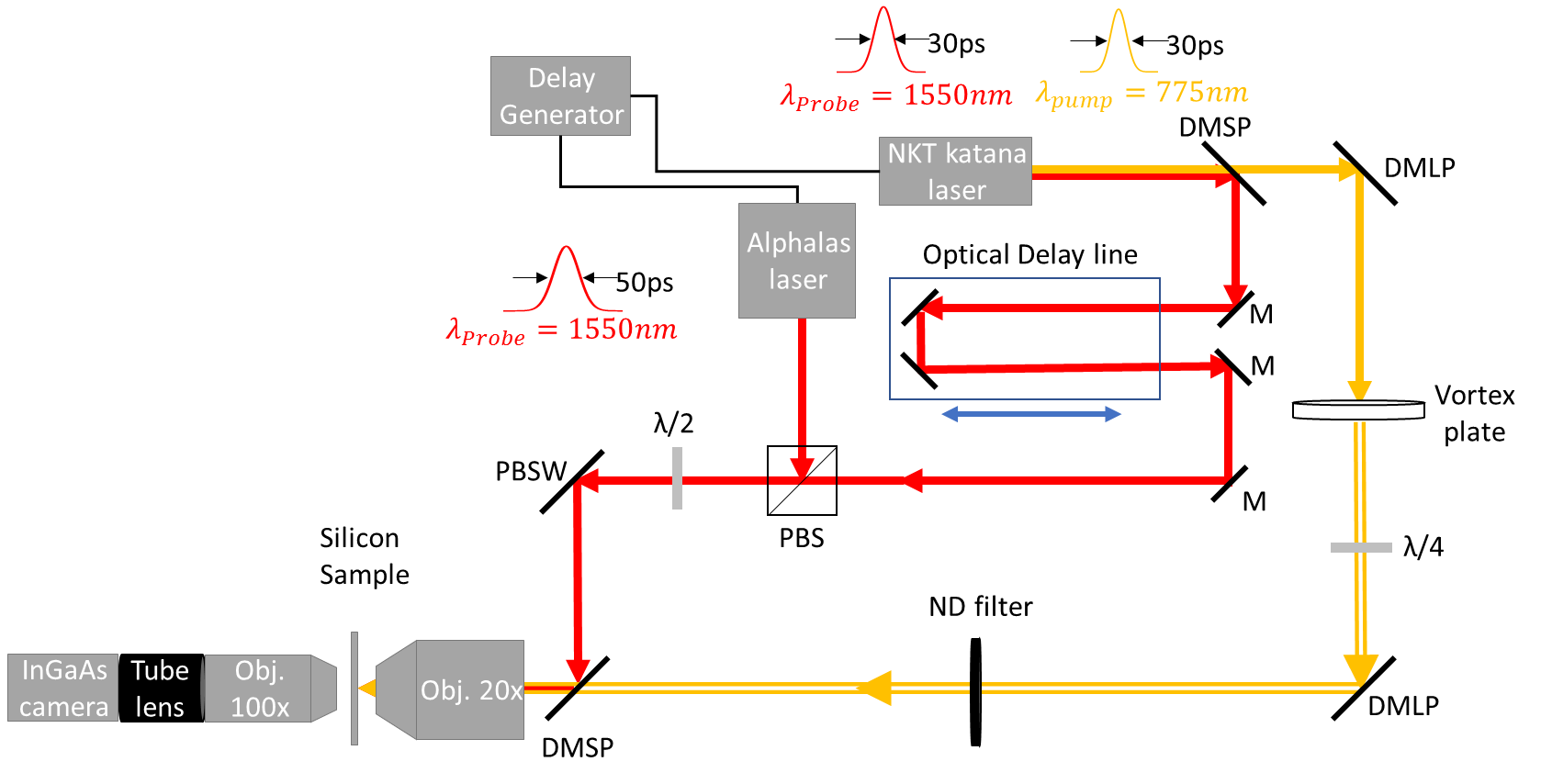


Fig. 1: Experimental setup, PBSW-Polarizing Plate Beamsplitter (PBSW-1550, Thorlabs), PBS-Polarizing Beamsplitter Cube (PBS124, Thorlabs), DMLP-Longpass Dichroic Mirror (DMLP950 , Thorlabs), DMSP-Shortpass Dichroic Mirror (DMSP950 , Thorlabs), M-Mirror (BB1-E04, Thorlabs), Obj. 20x (Mitutoyo M Plan Apo NIR 20x NA 0.4), Obj. 100x (Mitutoyo M Plan Apo NIR 100x NA 0.5), Vortex plate (RPC PHOTONICS VPP-m780)
